# Potential Role and Utilization of Plant Growth Promoting Microbes in Plant Tissue Culture

**DOI:** 10.3389/fmicb.2021.649878

**Published:** 2021-03-29

**Authors:** Abdoulaye Soumare, Abdala G. Diédhiou, Naveen Kumar Arora, Laith Khalil Tawfeeq Al-Ani, Mariama Ngom, Saliou Fall, Mohamed Hafidi, Yedir Ouhdouch, Lamfeddal Kouisni, Mame Ourèye Sy

**Affiliations:** ^1^AgroBioSciences Program, Mohammed VI Polytechnic University (UM6P), Ben Guerir, Morocco; ^2^Laboratoire Commun de Microbiologie (LCM) IRD/ISRA/UCAD, Centre de Recherche de Bel Air, Dakar, Senegal; ^3^Centre d’Excellence Africain en Agriculture pour la Sécurité Alimentaire et Nutritionnelle (CEA-AGRISAN), UCAD, Dakar, Senegal; ^4^Département de Biologie Végétale, Faculté des Sciences et Techniques, Université Cheikh Anta Diop (UCAD), Dakar, Senegal; ^5^Department of Environmental Science, School of Earth and Environmental Sciences, BBA University, Lucknow, India; ^6^Department of Plant Protection, College of Agriculture Engineering Sciences, University of Baghdad, Baghdad, Iraq; ^7^School of Biology Science, Universiti Sains Malaysia, Penang, Malaysia; ^8^Laboratoire Campus de Biotechnologies Végétales (LCBV), Département de Biologie Végétale, Faculté des Sciences et Techniques, UCAD, Dakar, Senegal; ^9^Laboratory of Microbial Biotechnologies, Agrosciences and Environment, Faculty of Sciences Semlalia, Cadi Ayyad University, Marrakesh, Morocco

**Keywords:** plant tissue culture, phytohormones, plant growth promoting microbe, agriculture, biotechnology, plant growth promoting bacteria, plant growth promoting fungi

## Abstract

Plant growth promoting microbes (PGPMs) play major roles in diverse ecosystems, including atmospheric nitrogen fixation, water uptake, solubilization, and transport of minerals from the soil to the plant. Different PGPMs are proposed as biofertilizers, biostimulants, and/or biocontrol agents to improve plant growth and productivity and thereby to contribute to agricultural sustainability and food security. However, little information exists regarding the use of PGPMs in micropropagation such as the *in vitro* plant tissue culture. This review presents an overview of the importance of PGPMs and their potential application in plant micropropagation. Our analysis, based on published articles, reveals that the process of *in vitro* classical tissue culture techniques, under strictly aseptic conditions, deserves to be reviewed to allow vitroplants to benefit from the positive effect of PGPMs. Furthermore, exploiting the potential benefits of PGPMs will lead to lessen the cost production of vitroplants during micropropagation process and will make the technique of plant tissue culture more efficient. The last part of the review will indicate where research is needed in the future.

## Introduction

Plant tissue culture consists of producing, under aseptic conditions, a whole plant from an explant or even a single plant cell. This component of plant biotechnology relies on the phenomenon of cell totipotency, which is the ability of any single cells to produce all the differentiated cells characteristic of organs, and to regenerate into an entire plant (Trigiano and Gray, 2016). Micropropagation exploits this fundamental property of plant cells for the rapid mass multiplication of elite’s genotypes on large scales in a comparatively short period of time. Nowadays, micropropagation plays a considerable role in agriculture, horticulture, and industry through the production of healthy seedlings throughout the year, and the reduction of the vegetal cycle (Suman, 2017). It is also a core technology for conservation of plant genetic resources, crop improvement, and propagation of new varieties from somaclonal variation, and genetic manipulation. However, the technique requires the use of chemical disinfectants, variable concentrations of appropriate phytohormones, and sometimes antibiotics, antifungals, antivirals almost at each stage of growth and development process (Liang et al., 2019). Some of these plant growth regulators (PGRs) are very costly, and therefore, limit or restrain the expansion of this technology and its agricultural profitability.

To implement this technology and reduce the cost-intensive process, plant growth promoting microbes (PGPMs) can be used as a sustainable solution (Verma et al., 2019). Indeed, many PGPMs can synthesize phytohormones and various other organic compounds which can improve plant growth and productivity. Different microorganisms, including bacteria, archaea, and fungi, living in the plant rhizosphere and feeding on sloughed-off plant cells and the proteins and sugars released by roots (Hütsch et al., 2002; Al-Ani, 2019a,b), have been used as PGPMs in agriculture. Unfortunately, PGPMs are not sufficiently used in *in vitro* plant tissue culture, and only few studies have reported inoculation with PGPMs in micropropagation (Srivastava et al., 2002; Souza et al., 2015; Lopes et al., 2019). Because the presence of microorganisms in the *in vitro* environment was almost universally perceived as negative for *in vitro* plant culture (Orlikowska et al., 2017), most of the research dealing with micropropagation and microbes focused on the detection and elimination of contaminants. However, many PGPMs can help in rooting, shoot elongation, and they can be useful in the success of acclimatization phase. Indeed, they can protect against biotic and abiotic stress that occurs *in vitro* propagation mainly at the hardening and acclimatization phase; two crucial steps for the success of micropropagation. PGPMs are key components for achieving sustainable agriculture, and, therefore, fostering the use of PGPMs in micropropagation is challenging.

This review aims to highlight the potential role of PGPMs in *in vitro* plant tissue culture, with special emphasis on micropropagation. The possible contributions of PGPMs in the advancement of agricultural crop production and the current constraints of their use will be emphasized and discussed.

## Plant Tissue Culture Technology

**Figure 1:**
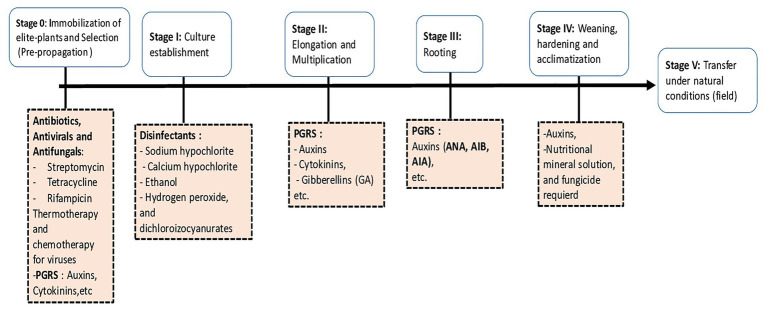
Main stages of micropropagation and required chemical components for each step.

Micropropagation is an *in vitro* culture technique which allows the mass multiplication of a plant material from a plant segment named explant. The explant may consist of any part of the plant such as an immature embryo, a seed, a portion of leaves, roots, or shoots, an anther, a pollen grain, an ovule, a meristem, or an apex. Micropropagation of plants also means the process of using explants and allowing them to undergo growth of undifferentiated or differentiated cells (Bidabadi and Jain, 2020). The explant is grown in a culture container filled with an artificial nutrient culture medium under sterile conditions. In addition to mass multiplication of elite plants, plant tissue culture technology also provides the means to multiply and regenerate novel plants from genetically engineered cells. This technology improves cultures by producing somaclonal and gametoclonal variants (Suman, 2017). The process of micropropagation can be divided into six stages (Figure 1):

Stage 0: Plant stock immobilization and pre-treatments, selection of the explant.
Stage I: Culture establishment.
Stage II:  Elongation and multiplication.
Stage III: Rooting.
Stage IV: Weaning, hardening, and acclimatization.
Stage V:  Transfer under natural conditions (to the field).

The first four stages of the micropropagation process generally take place in a highly protected environment without the possibility of interaction with microbes normally found in nature (Orlikowska et al., 2017). Therefore, the regenerated plants are vulnerable when transferred directly to field conditions. So, it is important to consider the interaction with beneficial microbes such as symbiotic and non-symbiotic fungi and bacteria. In recent years, some scientific works have attempted to re-establish this link with beneficial microorganisms in the process of *in vitro* multiplication. These microorganisms can positively impact the growth of explants and ensure better survival by sustaining the transplantation shock into greenhouse or glasshouse and in field conditions (Weyens et al., 2009; Srinivasan et al., 2014).

## Plant Growth Promoting Microbes and Their Multi-Functional Traits

**Figure 2:**
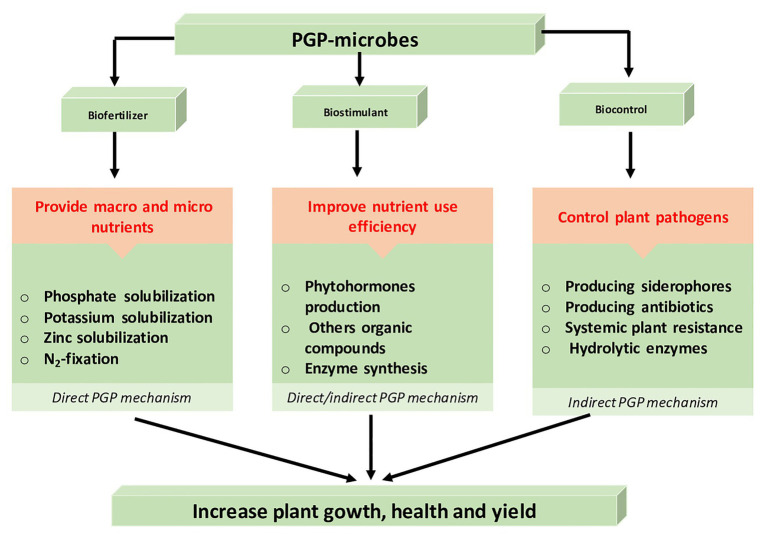
Role and mechanisms of rhizospheric plant growth promoting microbes.

Several soil microorganisms belonging to very different taxa have been identified as efficient PGPMs. Rhizospheric PGPMs are soil borne, living on root surfaces or colonizing the internal tissues of plants (named PGP endophytes) where they play different functions such as mineral solubilizing (Zn, P, and K), iron chelation, nitrogen fixation, production of phytohormones, and biocontrol ability against plant pathogens. Based on their activities, they are classified into three main groups corresponding to three growth promotion mechanisms (Figure 2):

- Biofertilizers, they increase the availability of nutrients and their utilization by plants.
- Biostimulants or phytostimulants, produce beneficial substances such as PGRs, which are not nutrients, pesticides, or soil improvers.
- Biocontrol agents, they control pathogens development through the production of antimicrobial metabolites or competition for space and nutrients.

Some of PGPMs can display two to three plant growth promoting mechanisms. Through their multi-functional roles, PGPMs influence all aspects of plant life including seed germination, nutrition, growth, and response to biotic and/or abiotic stresses (Weyens et al., 2009; Subramaniam et al., 2020a,b; Sunita et al., 2020). PGPMs may enhance plant growth and protection by direct and/or indirect modes of action. The direct mechanisms enhance plant growth either by providing nutrients or by producing growth regulators, while indirect mechanisms help the plant to grow healthily under abiotic stresses or protect the plant against infections, parasites, or certain predators (biotic stresses; Goswami et al., 2016; Arora et al., 2020).

There are two main groups of PGPMs: plant growth promoting fungi (PGPF) and plant growth promoting bacteria (PGPB). Plants establish a variety of interactions with soil fungi. Diverse taxa, belonging to arbuscular mycorrhizal fungi (e.g., *Gigaspora*, *Funneliformis*, and *Rhizophagus*), orchid mycorrhizal fungi (*Russula*, *Rhizoctonia*, and *Tulasnella* species), ericoid mycorrhizal fungi (*Harpophora oryzae* and *Colletotrichum tofieldiae*), ectomycorrhial fungi (e.g., *Laccaria*, *Pisolithus*, and *Scleroderma*), *Trichoderma* spp., *Piriformospora*, and other root endophytes fungi such as *Fusarium* spp., *Penicillium* spp., *Aspergillus* spp., etc., have been recognized as PGPF. Arbuscular mycorrhizal fungi (AMF) establish symbioses with over 90% of all plant species and influence host plants at various growth stages (Begum et al., 2019). While orchid mycorrhiza (ORM) and ericoid mycorrhiza (ERM) occur in specific plant lineages, i.e., Orchidaceae and Ericaceae subfamilies, respectively, (Martin et al., 2016; Perotto et al., 2018). Ectomycorrhizal fungi (EMF) are associated with 10% of plant families and are the dominant group in temperate and boreal forests, where they play a major role in the biology and ecology of forest trees (Smith and Read, 2008). PGPF infect plants without causing symptoms and express different lifestyles (mutualistic, latent pathogen, and latent saprophyte) depending on host genotype, age, and physiology. However, a small proportion of fungi are latent pathogens (Promputtha et al., 2007; Zabalgogeazcoa, 2008; Yuan et al., 2010).

**Figure 3:**
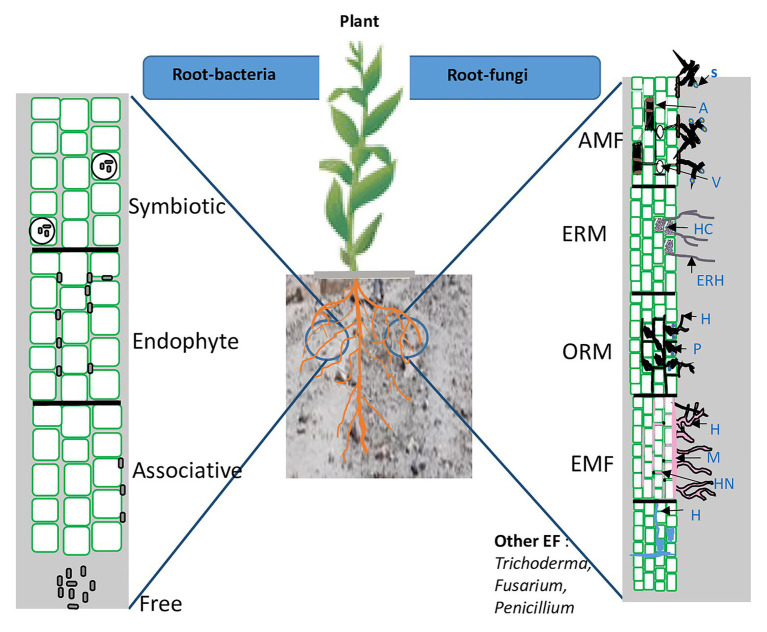
Schematic representation of root and rhizosphere colonization by beneficial microorganisms. AMF, arbuscular mycorrhizal fungi; ERM, ericoid mycorrhizal fungi; OMF, orchid mycorrhizal fungi; EMF, ectomycorrhizal fungi; EF, endophyte fungi; A, arbuscules; ERH, extraradical hyphae; V, vesicles; S, spore; HC, hyphal coils; P, peloton; HN, Hartig net; and M, mantle.

On the other hand, the PGPB group represent 2–5% of rhizospheric bacteria (Antoun and Prévost, 2005; Jha et al., 2010; Arora, 2015). They belong to the four bacterial groups including free-living bacteria, associative, endophytic bacteria, and nodule-forming bacteria (symbiotic). Like PGPF, they can act as biofertilizers, biostimulants, and/or biocontrol agents. The most widely exploited groups of PGPB belongs to genera of *Pseudomonas*, *Bacillus*, *Azospirillum*, *Azotobacter*, *Rhizobium*, *Bradyrhizobium*, *Frankia*, *Burkholderia*, *Thiobacillus*, *Serratia*, and *Streptomyces* (Adesemoye et al., 2008; Sivasakthivelan and Saranraj, 2013; Verma et al., 2019; Subramaniam et al., 2020a,b). The different interactions between the roots and surrounding soil PGPMs are summarized in Figure 3.

## Plant Growth Regulators Required in Micropropagation Process

Plant growth regulators are organic compounds synthetized within plants in response to specific stimuli and occur in extremely low concentrations. These chemical messengers or signal molecules play critical roles in regulating and controlling growth, development, reproduction, and senescence of the plant. In other hand, they control all aspects of plant development, from embryogenesis (Méndez-Hernández et al., 2019), regulation of organ size, defense against pathogens (Shigenaga and Argueso, 2016), stress tolerance (Ku et al., 2018; Ullah et al., 2018), and reproductive development (Pierre-Jerome et al., 2018). PGRs allow the plant to adapt to changing environments, by mediating growth, development, and nutrient allocation (Dias, 2019). Based on their origin, PGRs are divided into three groups: synthesized by plants, microbial origin, and synthetic compounds (Blázquez et al., 2020). Nowadays, the term of “phytoregulators” is used for both synthetic and natural organic PGRs. Five main classes of PGRs can be distinguished based on their chemical structures and effects: (i) auxins, (ii) cytokinins, (iii) gibberellins, (iv) abscisic acid, and (v) ethylene. Beside these classical plant hormones, other PRGs such as polyamines, analogs of diphenyl urea, salicylic acid, jasmonates, sterols, brassinosteroids, strigolactones oligosaccharins, phosphoinositosides, systemins, and florigen were discovered more recently. Among all PGRs, auxin and cytokinin classes are usually considered to be the most important phytohormones in plant growth regulation because they regulate many metabolic processes (Pour et al., 2019). PGPMs have the potential to produce these two hormones. Table 1 summarizes the different auxin and cytokinin compounds commonly used in plant micropropagation.

**Table 1:** Natural and synthetic auxin and cytokinin hormones commonly used in the micropropagation process of plants.

Some natural and synthetic **auxins** commonly used	Some natural and synthetic **cytokinins** commonly used
Indolyl-3-acetic acid (**IAA**)^*^	4-Hydroxy-3-methyl-trans-2-butenylaminopurine (**Zeatin**)^*^
Indolyl-3-butyric acid (**IBA**)^*^	6-Furfurylaminopurine (**Kinetin**)
	N6-(2-isopentyl) adenine (**2-iP**)^*^
2,4-Dichlorophenoxyacetic acid (**2,4-D**)	6-Benzylaminopurine or benzyl adénine (**BAP or BA**)
1-Naphthalene acetic acid (**NAA**)	

* Natural hormones.

## Plant Growth Promoting Microbes in Plant Tissue Culture

Traditionally, plant tissue culture systems are brought up in aseptic conditions. Thus, during the establishment of *in vitro* cultures, the explant is surface sterilized to eliminate all microorganisms. Since the role of PGPMs in plant growth and protection has been established, more attention has been paid to beneficial effects of these microorganisms in *in vitro* plant tissue cultures. In this respect, the use of competent PGPMs in micropropagation under *in vitro* and *ex vitro* conditions was analyzed and called “biotization” (Nowak, 1998). Microplant biotization is a biotechnological practice aimed at reducing chemical input in plant production (Kanani et al., 2020). The biotization can be done at all stages of *in vitro* propagation. In stage II and III of micropropagation by micro-cutting, PGPMs act generally as bio-stimulants by promoting elongation and increasing rooting, respectively, while in stage IV, they act as biocontrol agents and help to deal with biotic and abiotic stress factors (Figure 2). It is at this stage of acclimatization that biotization of microplants seems to be most important (Orlikowska et al., 2017). In addition to their three-main growth promoting mechanisms, certain PGPMs such as *Rhizobium*, *Frankia*, *Bradyrhizobium*, and mycorrhizal fungi have been recognized to be able to improve the physical properties of the soil by making it more conducive (Azcón-Aguilar and Barea, 2015; Egamberdieva et al., 2019).

### Beneficial Effects of Plant Growth Promoting Fungi in *in vitro* Plant Culture

Plants from micropropagation are adversely affected by water stress, because of low absorption capacity of their roots. Inoculation with AMF *in vitro* is an important tool to deal with this problem (Rai, 2001). Through biosynthesis of phytohormones or PGRs, AMF impact on post-transplant performance of *in vitro* grown plants by increasing nutrients availability and inducing resistance to pathogens (Rai, 2001; Akin-Idowu et al., 2009). According to Chanclud et al. (2016), Kemler et al. (2017), and Streletskii et al. (2019), fungi produce phytohormones such as auxins, cytokinins (CKs), abscisic acid (ABA), gibberellic acids (GAs), ethylene (ET), salicylic acid (SA), and jasmonic acid (JA). These hormones control plant development and activate signaling pathways during biotic and/or abiotic stresses. Meixner et al. (2005) showed that, plants inoculated with AMF had a higher level of auxins than non-inoculated plants. It has been shown that a large diversity of fungal species can produce CKs for hyphal development and nutrient uptake during mycorrhizal symbiosis. Auxin and cytokinin act as messengers to regulate various cellular processes in plants such as bud activity, branching, cell cycle, synchronization of fruit setting and dropping (Müller and Leyser, 2011), plant defense responses (Naseem and Dandekar, 2012), grain size, and biomass production (Osugi and Sakakibara, 2015). A balance of both auxins and cytokinins leads to the development of callus, i.e., a mass of undifferentiated cells.

In addition, fungi especially AMF play important role in water uptake and availability (Püschel et al., 2020), thereby increasing the rate of photosynthesis and osmotic adjustment under environmental stresses (Soumare et al., 2015a). AMF also increase the uptake of micronutrients such as P, Zn, Cu, Fe, etc. AMF contribution is especially important during the acclimatization phase because the adventitious and weak root system, without root hair, of vitroplants do not allow optimal absorption of nutrients from the soil during the early stage of the weaning step. AMF can help to overcome this problem, through their arbuscules and hyphae which transfer nutrients, especially phosphate from the soil to the plant (Karandashov and Bucher, 2005; Chen et al., 2018). Beneficial endophytic fungi promote plant growth by improving uptake of phosphorus, potassium, and zinc and/or production of phytohormones such as cytokinins, indole acetic acids, and gibberrellic acids (Rana et al., 2019). The lower survival rate and poor establishment of vitroplants in field conditions may be due to the fact that the transferred vitroplants did not find their natural microsymbiont partner. In this respect, Varma and Schuepp (1994) have shown that hydrangea vitroplants inoculated with the AMF, *Glomus intraradices* (current name: *Rhizophagus intraradices*) were strongly mycorrhized at the acclimatization stage and, therefore, the survival rate was 100% and no apparent “transient transfer shock” was visualized. Díez et al. (2000) showed that *in vitro* mycorrhization with *Pisolithus tinctorius* and *Scleroderma polyrhizum* strains increased the formation of secondary roots and the survival after acclimatization of cork oak vitroplants raised from somatic embryos. Similarly, Sahay and Varma (2000) reported a 90% post-transplantation survival rate of micropropaged tobacco and brinjal plants treated with the endophytic fungus, *Piriformospora indica*. This biopriming has also been reported to increase resistance against pathogen attacks (Harish et al., 2008). Reports of some successful biotization with endophytic fungi are enlisted in Table 2. Nevertheless, certain endophytic fungi can be plant pathogens and limit the micropropagation process. It is the case with *Fusarium equiseti* which was suspected to cause bamboo blight and culm rot disease (Tyagi et al., 2018).

**Table 2:** Growth regulators produced by microorganisms, and their effect on plant development and morphology.

Bacteria/fungi	Microbial phytohormones	Observed effects on explant	References
*Bacillus megaterium* MiR-4	Auxins	Root elongation [*Vigna radiata* (L.) R. Wilczek]	Ali et al. (2009)
*Pseudomonas putida*	Auxins (IAA, IBA, and NAA) Cytokinins (BA or BAP; 2iP, KN, ZEA)	Enhances biomass and essential oil production (*Mentha piperita*) Enhance resistance to osmotic stress (*Pennisetum glaucum Zea mays*)	Santoro et al. (2015) and Patel and Saraf (2017)
*Azospirillum brasilense* Sp245, SR80, and *A. halopraeferens*	Auxins (IAA)	Increases the effectiveness of clonal micropropagation of potato (*Solanum tuberosum* L.)	Vettori et al. (2010) and Kargapolova et al. (2020)
*A. brasilense* spp.	Auxins	Root elongation and sprouts, number of roots (*Arabidopsis thaliana*)	Vega-Celedon et al. (2016)
*Pseudomonas* sp., *Bacillus* sp.	Auxins (IAA)	Root elongation [*Arabidopsis thaliana* (L.) Heynh.]	Asari et al. (2017)
*Arthrobacter*, *Bacillus*, *Azospirillum*, and *Pseudomonas*	Cytokinins (IBA and NAA)	Stimulated root biomass of *Platycladus orientalis*	Naz et al. (2009) and Liu et al. (2013)
*Azospirillum lipoferum*	Gibberellins (GA3)	Elongate the stem and shoots of *A. glutinosa*	Gutierrez-Manero et al. (2001)
*Bacillus amyloliquefaciens*	Gibberellins (GAs)	Improved rice (*Oryza sativa* L.) plant growth	Shahzad et al. (2016)
*Azospirillum brasilense*	Abscisic acid (ABA)	Help in plant-stress alleviation in *Arabidopsis thaliana*	Cohen et al. (2015)
*Streptomyces sp. strain DBT204*	IAA and Kinetin (KN)	Enhancing growth of chili and tomato seedlings	Passari et al. (2016)
*Fusarium* strain	Auxin	Significant increase in growth and all tested growth parameters for *Euphorbia pekinensis*	Dai et al. (2008)
Ectomycorrhizal fungi (*Astraeus odoratus*, *Gyrodon suthepensis*, *Phlebopus portentosus*, *Pisolithus albus*, *Pisolithus orientalis*, and *Scleroderma suthepense*)	IAA	Increase the elongation of rice and oat (*Avena fatua* L.) coleoptiles	Kumla et al. (2020)

CK, cytokinin; GB, gibberellin; IAA, indole-3-acetic acid; BAP, benzylaminopurine; ABA, abscisic acid; KN, kinetin; IBA, indole-3-butyric acid; NAA, naphthalene acetic acid; and ZEA, zeatin.

Although, the mycorrhization technique is important for the growth and development of the micropropagated plantlets, some problems need to be solved to optimize the technology efficiency. The main problem to be solved is how to produce pure fungal inoculum without contaminants for micropropagation. Currently, the disinfection and germination of spores in the agar medium are difficult. On the other hand, Murashige and Skoog (1962) medium (MS) systematically used in micropropagation does not seem to be favorable for the germination and growth of spores (Rana et al., 2019). This suggests that the methodology of propagation needs to be adapted by modifying the nutrient medium to overcome the problem. To overcome the obligatory biotrophy of AMF, the production of axenic inoculum from *Ri* T-DNA transformed carrot roots, under elevated CO_2_, raised great hope (Srinivasan et al., 2014). This method allowed significant production of extensive hyphal growth on modified Strullu Romand (MSR) medium and 8,500–9,000 spores per petri dish (Srinivasan et al., 2014). The possible utilization of sonication, gradient flotation, and enzymatic methods to separate intraradical spores and vesicles from roots and thereby to obtain a high-quality inoculum has been pointed out by Biermann and Linderman (1983). However, these processes seem to be time consuming, costly, and tedious.

### Beneficial Effects of Plant Growth Promoting Bacteria in *in vitro* Plant Culture

The first *in vitro* bacterization was reported by Digat et al. (1987). These researchers showed a positive impact of *Pseudomonas putida* and *Pseudomonas fluorescens* strains on the rooting and acclimatization of *Primula microshoots*. A few years later, Elmeskaoui et al. (1995) have shown that biotized plant tissue cultures benefit from microbial presence through an improvement in photosynthetic efficiency and biomass production. Generally, PGPB improve growth by releasing PGRs required for vitropropagation (Quambusch and Winkelmann, 2018). Auxins and cytokinins biosynthesis are widespread among rhizobacteria, and different biosynthesis pathways have been identified (Kado, 1984; Amara et al., 2015). For instance, it is assumed that many bacteria can produce cytokinins in pure culture and more than 80% of soil bacteria in the rhizosphere can produce auxins especially indole-3-acetic acid (IAA) which is the major auxin active form in plants (Patten and Glick, 1996; Souza et al., 2015). PGRs from microorganisms play a compensatory role, especially when micropropagated plants are under sub-optimal environment with insufficient endogenous production. For different strains belonging to *Bacillus*, *Pseudomonas*, *Rhizobium*, *Bradyrhizobium*, *Enterobacter*, *Methylobacterium*, *Microbacterium*, *Rhodococcus*, and *Acinetobacter*, these PGRs have been quantified, characterized, and tested in plant tissue culture (Spaepen and Vanderleyden, 2011). Vereecke et al. (2000) identified 11 different cytokinins in the supernatant of the culture medium of *Rhodococcus fascians*. Their application *in vivo* and *in vitro* on the plant leads to galls, stem fasciation, and brooms. The study from Erturk et al. (2010) has demonstrated that different PGPB strains belonging to genus *Bacillus*, *Paenibacillus*, and *Comamonas* promoted root formation in kiwifruit cuttings in mass clonal propagation through IAA production. More recently, Lim et al. (2016) reported that the diazotroph *Herbaspirillum seropedicae* induced the proliferation and differentiation of calli and embryogenic calli of oil palm through nitrogen fixation and IAA production. Similar findings were previously reported by Rodríguez-Romero et al. (2008) on micropropagated banana plants with *P. fluorescens*, with a consistent increase of plant development. Kargapolova et al. (2020) have shown the efficacy of the inoculation with *Ochrobactrum cytisi* on potato microplants. A 50% increase of mitotic index of root meristem cells and 34% increase of shoot length were reported under *ex vitro* conditions. On the other hand, some plant growth promoting rhizobacteria (PGPR) can induce the production of phytohormones by the plant. Analyzing plant molecular responses to *Burkholderia phytofirmans* colonization, Poupin et al. (2013) showed that genes involved in auxin and gibberellin pathways were induced in *Arabidopsis thaliana*. Moreover, bacterial phytohormones such as gibberellins can interact with other hormones to support elongation (Bottini et al., 2004). Other phytoregulators such as abscisic acid and salicylic acid are produced by PGPB, but they are less studied. The phytohormones regulate both growth and senescence by modulating ethylene levels in the plant tissue (Khan et al., 2008; Iqbal et al., 2017). The later plays an essential role in the plant defense mechanisms against infections and external aggressions. Decreasing ethylene levels allows the plant to be more resistant to different environmental stresses (Glick, 2005). The findings of Nowak (1998) revealed that *Origanum vulgare* (L) plantlets inoculated with *Pseudomonas* spp., produced more phenolic compounds and chlorophyll than non-inoculated plantlets. Phenolic compounds are involved in plant pigmentation, growth, reproduction, and resistance to pathogens (Lattanzio et al., 2006). The growth of pathogens is suppressed by producing toxins, antibiotics, HCN, and/or hydrolytic enzymes such as proteases, chitinases, and lipases. These compounds degrade the cell wall, virulent, or pathogenic factors (Compant et al., 2005). It has been shown that inoculation with PGPB that produced aminocyclopropane carboxylate (ACC) deaminase enhanced stress tolerance and plant growth through a reduction of ethylene production (Ruzzi and Aroca, 2015; Gupta and Pandey, 2019). Souza et al. (2013) reported that ethylene acts as stress phytohormone which adversely affects the growth of the roots under abiotic and biotic stress. Similar results were previously reported on *Camelina sativa* by Heydarian et al. (2016). These authors have shown that PGPB can enhance growth and salt tolerance in camelina by the production of ACC deaminase. *In vitro* co-culture of explants with PGPB induces developmental and metabolic changes, which enhance their tolerance to abiotic and biotic stresses. In this regard, Sgroy et al. (2009) have demonstrated that *Bacillus*, *Lysinibacillus*, *Pseudomonas*, *Achromobacter*, and *Brevibacterium* associated with the halophyte *Prosopis strombulifera* act as stress homeostasis-regulating bacteria through IAA, zeatin, and GA production. These phytohormones increase roots length of *P. strombulifera*, allowing them to explore the soil in depth and absorb more water. In hydroponic media, De Garcia Salamone et al. (2005) compared *Phaseolus vulgaris* plants treated with auxin and cytokinin to those inoculated with Rhizobium. They noticed that the results in terms of growth were similar and the PGRs were detected in the medium of inoculated plants but not in the medium of non-inoculated roots. Bacteria can also produce volatile metabolites which can induce organogenesis (Gopinath et al., 2015), improve the efficiency of photosynthesis (Xie et al., 2009), and provide protection against abiotic stressors (Orlikowska et al., 2017). PGP bacteria and fungi living in the rhizosphere induce systemic resistance (ISR) and enhance defense against a broad range of pathogens and insects. Some PGPB (e.g., *Pseudomonas* and *Bacillus*) as well as some PGPF (e.g., *Trichoderma*) can sensitize the plant immune system for enhanced defense without directly activating costly defenses (Pieterse et al., 2014; Al-Ani and Mohammed, 2020).

## Application in Agriculture, Horticulture, and Forestry

The development of biotization is a promising avenue which is gaining increasing amounts of attention from researchers in agriculture, horticulture, and forestry.

**Figure 4:**
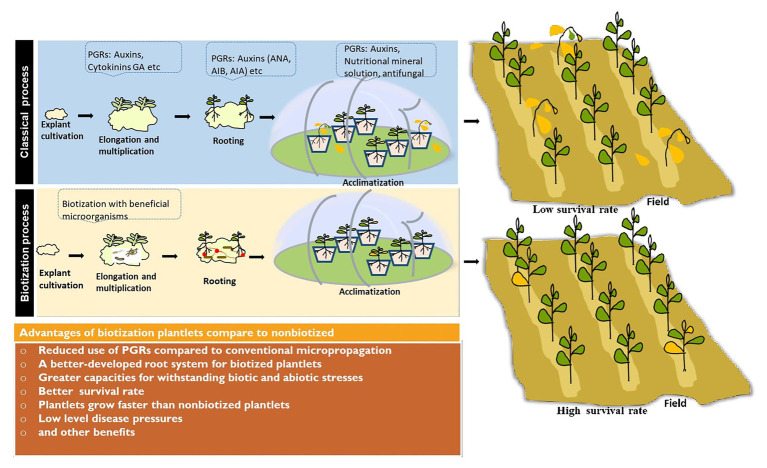
Schematic summary of some benefits of biotization process compared to classical micropropagation process.

Plant tissue culture is considered as one of the important breeding methodologies and an efficient way of clonal propagation allowing to increase production of important crops (El-Sherif, 2018) such as groundnut or peanut (*Arachis hypogaea* L.), sorghum (*Sorghum bicolor*), carrot (*Daucus carota* L.), potato (*Solanum tuberosum*), maize (*Zea mays*), wheat (*Triticum aestivum* L.), and rice (*Oryza sativa*). Rice is one of the most important crops which currently feeds more than 50% of the world’s population (Ricepedia, 2020). Therefore, various protocols have been developed for rice plant tissue culture, but high mortality of micropropagated plants during or following the transfer from laboratory to land is still a limiting factor. In order to increase growth and reduce mortality rate in plantlets at the acclimatization stage, introduction of beneficial microorganisms (bacteria and/or fungi) was suggested (Srivastava et al., 2002). Indeed, some studies reported that endophytic fungi such as *Phialemonium dimorphosphorum*, *Gaeumannomyces graminis*, and *Gaeumannomyces amomi* significantly increase rice plant height, root length, and root system development (Kandar et al., 2018). Similary, Diédhiou et al. (2016) reported that the inoculation of rice plants with AMF can significantly increase plant biomass and grain yield of certain varieties under field conditions. Furthermore, Bernaola and Stout (2020) reported that AMF colonization influences the resistance of rice plants to herbivore feeding or pathogen infection. Biotization of micropropagated rice plants results in enhanced growth and higher survival rate during laboratory to land transfer (Chandra et al., 2010). Senthilkumar et al. (2008) reported that the biotized rice plants performed better, for root and shoot length, biomass, and grain yield over the uninoculated control. Other successfully biotization experiments were reported with *Azotobacter chroococcum*, which increased the shoot weight and number of roots in wheat (Andressen et al., 2009), *Azospirillum barasilense* which enhanced acclimatization of micropropagated fruit rootstocks (Vettori et al., 2010), and *Pseudomonas aureofaciens* which leaded to a better growth of potato and strawberry at acclimatization, when microshoots were inoculated before rooting (Zakharchenko et al., 2011). A pseudomonas strain has been also reported to promote root growth of watermelon (Nowak, 1998). Recently, our results showed that *Streptomyces griseorubens* and *Norcardiopsis alba* increased maize rooting, root hairs, and growth under phosphorus deficiency (Soumare et al., 2020a). Figure 4 summarizes some benefits of biotization compared to classical micropropagation.

Among ornamental plants, orchids dominate among the commercially micropropagated, and attract more attention. Orchidaceae is one of the largest family of flowering plants and horticulturally important species due to their ornamental and commercial values. In addition, orchids are used as traditional medicines (Rahamtulla et al., 2020). *In vitro* culture is a useful method to propagate endemic or endangered orchid species for conservation purposes because seeds of most of these plants are difficult to germinate. In nature, the germination of orchid seeds is induced by specific mycorrhizal fungi (symbiotic germination) which promote the embryo growth and supply it with required nutrients (Liu et al., 2010; Herrera et al., 2020). An effective system for the *in vitro* propagation of orchids must integrate the associated symbiotic fungi through biotization approach. This will allow good micropropagation and acclimatization with no apparent “transient transfer shock.” In addition, the technique of biotization could be a realistic way to produce low-cost micropropagated plantlets (Bezerra et al., 2020). Some bacteria have also exhibited good potential for application in orchid cultivation. In this sense, Bezerra et al. (2020) reported positive impact of the application of rooting-derived microorganisms, especially bacterial isolate in the *in vitro* culture and plantlet acclimatization of *Oncidium varicosum*. *In vitro* bacterization of photinia (*Photinia* × *fraseri* Dress), another ornamental plant has yielded interesting results. Using the PGPB *Azospirillum brasilense* and *Azotobacter chroococcum* during rhizogenesis, Larraburu et al. (2007) have reported that they induced earlier rooting of photinia shoots, and significant increase of root fresh and dry weight and root surface area. These results are in agreement with previous findings from Digat et al. (1987) which showed a positive impact of *P. putida* and *P. fluorescens* strains on the rooting and acclimatization of microshoots of *Primula*, a genus including many important commercial ornamental species.

It is widely admitted that deforestation and forest degradation take place at a faster rate than they are being regenerated naturally or replanted artificially. The potential benefits of *in vitro* plant regeneration in afforestation and reforestation programs have long been recognized (Di-Gaudio et al., 2020). Biotization in micropropagation technology may add advantage in terms of cost production of tissue-cultured plantlets. In nature, all species of forest trees depend upon a symbiotic association of their roots with ecto and/or endomycorrhizal fungi (Brundrett, 2009). Moreover, it has been shown that trees often fail to establish at new sites if their mycorrhizal fungal symbionts are absent (Lakhanpal, 2000). Therefore, to maximize the benefits of micropropagation, it is necessary to allow the vitroplants to form effective mycorrhiza, especially for species with high mycorrhizal dependency. *In vitro* mycorrhization of micropropagated plants before acclimatization increases survival and resistance to water stress and ensures a better mineral nutrition of the plant by enhancing the functionality of the root system (Siddiqui and Pichtel, 2008). Numerous findings validate the use of *in vitro* mycorrhization techniques for several plants such as *Castanea sativa* (Martins, 2008), *Helianthemum* spp. (Morte et al., 1994), *Cistus* spp. (Quatrini et al., 2003), *Quercus suber* L. (Díez et al., 2000), and *Eucalyptus* (Freire et al., 2018; Di-Gaudio et al., 2020). Among these species, *Eucalyptus* genus is the most used species in forest plantation and in reafforestation programs (Soumare et al., 2017). *Eucalyptus* species are widely used for rapid production of solid wood and cellulose pulp (Soumare et al., 2015b, 2017). Unfortunately, micropropagated plants are adversely affected by water stress and require a long period of transition to become adapted to *ex vitro* conditions. Reddy and Satyanarayana (1998) showed that establishment of mycorrhiza in micropropagated plantlets of *Eucalyptus tereticornis* enables them to survive in *ex vitro* conditions more readily and improves their growth and acclimatization. Similarly, Nowak (1998) showed that *in vitro* mycorrhization of micropropagated plants has increased plant survival and shoot biomass during *ex vitro* weaning as well as to shorten the acclimatization phase. With very little modification, biotization approach can be widely applicable to other useful forest tree species used in reforestation programs, especially those with high mycorrhizal dependency (Diédhiou et al., 2004, 2005; Bâ et al., 2010). Niemi and Scagel (2007) have developed an *in vitro* micropropagation method to induce adventitious root formation in hypocotyl cuttings of Scots pine (*Pinus sylvestris*) by inoculating them with two ectomycorrhizal fungi, *Pisolithus tinctorius* and *Paxillus involutus*. In addition, the formation of their coat mantle on the external surface of the root, create a barrier against the soil borne phytopathogens.

## Future Prospects for Plant Growth Promoting Microbes in Plant Tissue Culture

The role of PGPMs in tissues cultures has not been studied sufficiently enough. Our knowledge in PGPM behavior at the root, leaf, or whole plant level and their function in the natural environment is still limited (Sunita et al., 2020). In the future, much research is needed to select efficient, multifunctional, stress tolerant PGRs-producing microbes and having ecological plasticity for their use in plant tissue culture. Indeed, the wide diversity of possible uses of beneficial microbes in plant tissue culture open new doors to identify appropriate candidates from PGPMs to be used at the different stages of micropropagation, with particular attention to mixed-strain consortiums rather than mono-strain inoculums to take advantage of functional complementarity (Soumare et al., 2020b). On the other hand, a great deal of effort should be devoted toward bioformulation of these microbes for suitable application in plant tissue culture. Currently, there is some constraints for the delivery of PGPMs, especially during explant cultivation, elongation and multiplication, and rooting. To implement their application of PGPMs in plant tissue culture, researchers should develop strategies to improve microbial inoculants and inoculation technologies. In this respect, the application of bionanotechnologies could provide new avenues for the development of carrier based microbial inoculants. The use of nanoformulations may enhance the stability of biofertilizers (Malusá et al., 2012; Arora et al., 2020) with respect to heat, desiccation, and UV inactivation. Currently, very few studies are interested in bionanotechnology inputs in *in vitro* tissue culture. Research would be based on what is done in the pharmaceutical industry in order to develop tailor bioformulations using PGPMs especially for the biotization purpose in plant tissue culture. The other challenge is improving the quality of microbial inoculants for vitroplants as well as developing adequate inoculation protocols. In this sense, the utilization of genetically modified inoculants may offer opportunities in order to achieve a specific purpose in the agricultural and/or food sector. Recent advances in biotechnological tools, such as functional genomics, signaling in rhizosphere, etc., could be useful in engineering of micro-organisms to confer improved benefits to plant especially in plant tissue culture field.

## Conclusion

Plant tissue culture technique constitutes an important tool in modern agriculture, horticulture, and forestry. However, the process is expensive due to the requirement of chemicals and the high rate of mortality during the acclimatization phase. PGPMs have the innate potential to produce PGRs and can be considered as potential biofactories. The development and use of inoculants based on PGPMs will help to lessen the cost production of vitroplants by partially or totally replacing some commercial synthetic products with microbial phytohormones and by increasing the survival rate of vitroplants. However, the use of microbes deserves careful monitoring of endophytic communities, especially for plants used as raw food because some pathogenic strains for humans can be stably maintained in cultivated tissues and *ex vitro* plants. In addition, much remains to be learned from PGPMS in order to identify appropriate candidates and to develop bioformulations for suitable application in plant tissue culture. Along this same line, works on the responses of crops and other useful plants to inoculation with symbiotic and non-symbiotic PGPMs will help to identify which plants are suitable candidates for the microplant biotization.

## Author Contributions

All authors listed have made a substantial, direct and intellectual contribution to the review and approved it for publication. AS and AD had the initial idea of the article, performed the literature search, and drafted the work. NA, LA-A, MN, SF, MH, YO, LK, and MS critically revised the paper.

### Conflict of Interest

The authors declare that the research was conducted in the absence of any commercial or financial relationships that could be construed as a potential conflict of interest.
